# Ocean warming and acidification may drag down the commercial Arctic cod fishery by 2100

**DOI:** 10.1371/journal.pone.0231589

**Published:** 2020-04-22

**Authors:** Martin C. Hänsel, Jörn O. Schmidt, Martina H. Stiasny, Max T. Stöven, Rudi Voss, Martin F. Quaas

**Affiliations:** 1 Sustainable Fishery, Department of Economics, Kiel University, Kiel, Germany; 2 Potsdam Institute for Climate Impact Research, Potsdam, Germany; 3 Pelagic Fish Research Group, Institute of Marine Research, Bergen, Norway; 4 Biodiversity Economics, German Centre for Integrative Biodiversity Research (iDiv) Halle-Jena-Leipzig, Leipzig, Germany; 5 Department of Economics, Leipzig University, Leipzig, Germany; Aristotle University of Thessaloniki, GREECE

## Abstract

The Arctic Ocean is an early warning system for indicators and effects of climate change. We use a novel combination of experimental and time-series data on effects of ocean warming and acidification on the commercially important Northeast Arctic cod *(Gadus morhua*) to incorporate these physiological processes into the recruitment model of the fish population. By running an ecological-economic optimization model, we investigate how the interaction of ocean warming, acidification and fishing pressure affects the sustainability of the fishery in terms of ecological, economic, social and consumer-related indicators, ranging from present day conditions up to future climate change scenarios. We find that near-term climate change will benefit the fishery, but under likely future warming and acidification this large fishery is at risk of collapse by the end of the century, even with the best adaptation effort in terms of reduced fishing pressure.

## Introduction

Achieving the sustainable development goal for the oceans requires a sustainable use of its renewable natural resources [1]. In this respect marine fisheries are not only essential for food security and protein intake [2], but also support the livelihoods of roughly 500 million people dependent on fisheries [3]. The increasing release of carbon dioxide (CO_2_) into the atmosphere causes global warming, while at the same time its dissolution in upper ocean waters leads to ocean acidification [4]. In addition to overfishing, both effects have been identified as potential major stressors to marine fish and subsequently fisheries. Ocean warming affects species distribution [e.g. 5–7] and vital rates, such as growth and mortality [8]. In the case of ocean acidification experimental work shows effects on behavior [9–12] and vital rates [12–14]. Although evidence for commercially exploited fish species is still sparse, it does suggest non-uniform reactions for different species or stocks [14–19].

Given the uncertainties on the single-fish species level it is by no means easy to up-scale physiological responses to ocean warming and acidification to the level of the fish population. This exercise, however, is essential to inform policy-makers about possible consequences for food security and help designing appropriate mitigation and adaption strategies. To this end, one possible methodology is to incorporate the effects of ocean warming and acidification into the stock-recruitment relationship within a bio-economic fishery model. This strand of literature is relatively new and has mostly analyzed either ocean warming [20–22] or ocean acidification [23–26] in isolation. For example, Årthun et al. [22] use a variety of temperature, salinity and climatic indices to predict Northeast Arctic cod recruitment. Only very few studies [27–30] combine ocean warming and acidification effects on recruitment within an integrated bio-economic model.

Lam et al. [27] feed a Dynamic Bioclimate Envelope Model with outputs of physical variables capturing ocean warming and acidification from four different Earth System Models to project future changes in the distribution and maximum catch potential of Arctic fisheries. Fernandes et al. [28] combine an ecological model that captures habitat suitability, species ecophysiology, population dynamics and metabolic theory with economic input-output analysis to study how ocean warming and acidification affect commercially important fisheries in the UK. Unlike Lam et al. and Fernandes et al., our model explicitly includes dynamic feedbacks between the ecological and the economic part of the model.

Closest to our modelling framework are Cooley et al. [29] and Voss et al. [30]. Cooley et al. [29] focus on the United States Atlantic sea scallop fishery by modelling ocean acidification effects on juvenile sea scallop as well as ocean warming and acidification effects on adults. OA effects on scallop recruitment are included into a Berverton-Holt stock recruitment relationship while deep-water temperature and ocean acidification effects on scallop growth are used to adjust the body growth coefficient of adult scallops, which is assumed directly proportional to the growth in calcification. Voss et al. [30] employ a methodology very similar to our study. They alter the parameters of growth and natural mortality of a Ricker type stock-recruitment function in a dynamic age-structured fishery model and find that the management of the Western Baltic Cod fishery will not be able to adapt to the combined effect of ocean warming and acidification.

Our contribution to this strand of literature is twofold: First, we provide a very relevant case study. The Northeast Arctic cod (*Gadus morhua*) is the world’s largest cod stock supporting one of the commercially most valuable fisheries with a long history [31–33]. The effect of rising temperatures on Northeast Arctic cod recruitment has so far been positive [21–22], and likely has supported stock management and assisted in stock recovery over recent years. However, comparisons between cod stocks suggest a non-linear relationship between temperature and recruitment. At bottom temperatures below 5°C recruitment increases with sea surface temperature, while recruitment decreases above bottom temperatures of 8.5°C [34–35]. Whereas overall the effects of ocean acidification are still less understood, there is evidence that ocean acidification may have a significant effect on recruitment of Northeast Arctic cod [14, 36]. It is therefore becoming increasingly important to not study the effects of ocean warming in isolation, but in interaction with ocean acidification as an additional stressor that impedes recruitment success. Moreover, due to polar amplification the Arctic Ocean and the marginal Barents Sea are warming faster than the global average [37, 38, 4] and are expected to experience the strongest acidification of the global ocean [39].

Second, our methodology brings together time-series econometrics, experimental data and bio-economic modelling. Thereby it offers a promising methodology to analyze the sustainability of renewable natural resources and its repercussions on key socio-economic indicators. Our results confirm the initial positive effect of ocean warming on the Northeast Arctic cod stock, but also indicate an optimal temperature beyond which additional warming negatively influences recruitment and fishing outcomes. When considering the additional effect of ocean acidification that is projected under a business-as-usual scenario, the fishery could be at risk of collapse by the end of the century, even if fishery management would optimally adapt fishing pressure and gear selectivity. Because Northeast Arctic cod supports one of the most important fisheries in the world, the stock’s contribution to sustainable food production could be at stake in a few decades’ time.

## Materials and methods

We quantify both the isolated and combined effects of ocean warming and acidification on Northeast Arctic cod recruitment using time-series data of ocean temperature from the Kola Section–one of the longest oceanic time-series and a well-known indicator of Barents sea temperature [40]–as well as published experimental data from Stiasny et al. [14] that show acidification to have a strongly negative effect on recruitment. We scale up physiological responses to ocean warming and acidification to population-level processes by considering how both stressors could modify the parameters of growth, mortality and reproduction within a Ricker type stock-recruitment relationship [41] in an age-structured fishery model. Following Hjermann et al. [42] and Röckmann et al. [43], our main assumption is that ocean warming and acidification both cause changes in early life stage mortality rates leading to a density-independent mortality rate during the recruitment phase.

Mortality during the recruitment process is due to density-independent and density-dependent effects, both of which may potentially be affected by warming and acidification. Although Myers and Cadigan [44] and Fromentin et al. [45] showed that density-dependent mortality can be important for cod populations in general, there is no evidence linking density-dependent mortality to changing environmental conditions.

### Population level effects of ocean warming

To include the effects of ocean warming in the stock-recruitment model for Northeast Arctic cod, we use annual time-series data on spawning stock biomass and recruitment numbers from 1946 to 2013 supplied by the International Council for the Exploration of the Sea [46]. The monthly temperature data were taken from the Kola Section [40]. Temperature data are the monthly-integrated mean for 0–200 m depth along the 33°30′E meridian from 69°30 to 77°00′N for each year between 1921 and 2013. Although these data are not depth-specific, the monthly-integrated mean is a widely used indicator for the impacts of ocean warming on cod populations in the Barents Sea [47–50]. Using Ordinary Least Squares regression, we find that the January temperature statistically best explains recruitment within this period.

Using *R*_*t*+3_ for the number of recruits in year *t* at age 3 years, SSB_*t*_ for spawning stock biomass in year *t*, *T*_*t*_ for the Kola Section in January of year *t*, and *ε*_*t*_ to denote an independent and identically distributed error term, we estimate the following stock-recruitment function:

$$\log\left(R_{t+3}/SSB_{t-1}\right)=\varphi_{0}-\varphi_{1}SSB_{t-1}+\varphi_{T}T_{t}+\varphi_{T^{2}}T_{t}^{2}+\varepsilon_{t}$$ (1)

The final estimators are given in Table 1.

**Table 1:** Estimates for coefficients of stock-recruitment model.

Parameter	Estimate	Lower bound (5%)	Upper bound (95%)	Unit
*φ*_0_	-7.59	-14.04	-1.13	
*φ*_1_	0.0014	0.0009	0.0019	(1000 tons) ^-1^
*φ*_*T*_	4.03	0.74	7.32	Celsius^-1^
$\varphi_{T^{2}}$	-0.45	-0.86	-0.037	Celsius^-2^

R^2^ = 0.39, F-statistic: 12.97, p-value <0.001.

We find a non-linear effect of the January seawater temperature on recruitment. Initially a one-percent increase in January seawater temperature leads a 4.03% increase of the 3-year old recruits per spawning stock biomass, while this effect decreases by 0.45% for every additional percentage increase in temperature. Hence, we find that stock recruitment for Northeast Arctic cod is an increasing but concave function of the January seawater temperature. The latter implies an optimal temperature beyond which additional warming has a negative effect on recruitment.

### Population level effects of ocean acidification

Estimates of mortality increase due to ocean acidification are based on experiments by Stiasny et al. [14] in which the survival of cod larvae was quantified in direct response to increased partial pressure of CO_2_ (*p*CO_2_) levels. Eggs and larvae from the Northeast Arctic cod stock caught in the Barents Sea were kept under control conditions (~400–500 μatm corresponding to *pH* 7.6±0.03) and elevated CO_2_ conditions (~1100 μatm corresponding to *pH* 7.9±0.15) until 25 days post-hatching, and survival was monitored closely. The elevated *p*CO_2_, which is globally predicted to be likely reached by the end of the century under the IPCC RPC8.5 scenario, but due to the increased solubility in colder waters and retreated sea ice in the Arctic is likely reached much sooner in the Arctic, resulted in a doubling of daily mortality rates compared to present-day CO_2_ concentrations during the first 25 days post hatching, a critical phase for population recruitment. The results were consistent under different feeding regimes. In this study we use the estimate of Stiasny et al. [14] in their more conservative scenario, implying that recruitment under end-of-century acidification may be reduced to 24.5% of the recruitment under baseline CO_2_ levels. While the time-series approach for integrating ocean warming effects allows us to model a continuum of ocean warming, the experimental results only support an analysis of the additional effect of projected end-of century ocean acidification compared to a scenario without acidification.

### Age-structured ecological-economic optimization model

Following the approach of Tahvonen et al. [51,52], we apply an ecological-economic optimization model for a fishery of an age-structured fish stock, parameterized for the Northeast Arctic cod fishery. The model calculates the economically optimal fishing effort and related total allowable catch (TAC) to be set under steady state conditions. This analysis considers maximum economic yield management, i.e. catch, gear choice and age-specific stock numbers are determined to maximize steady-state economic surplus. We use *x*_*st*_ to denote the number of fish in age class *s* = 1,…,*n* at the beginning of year *t*. The number of fish that are caught from age class *s* in year *t* is denoted by *h*_*st*_. We include *n* = 13 age classes with the last age class subsuming all individual fish that are 13 years or older. Spawning stock biomass follows as

$$SSB_{t}=\sum_{s=1}^{n}\;‍w_{s}g_{s}x_{st}$$ (2)

for which weights-at-age *w*_*s*_ are taken from ICES [46: table 3.8] and maturity-at-age *g*_*s*_ from ICES [46: table 3.11]. The relationship between spawning stock biomass and recruitment three years later is captured by calculating recruitment as one-year old fish that then face zero natural mortality until becoming three-year old fish. Because of this implementation strategy, the Ricker-type stock-recruitment function includes the new variable *x* for recruitment, while the estimates for *φ*_0_, *φ*_1_, *φ*_*T*_ and $\varphi_{T^{2}}$ remain as reported in Table 1.

$$x_{1,t+1}=\phi_{0}\;SSB_{t}\;e^{\varphi_{0}-\varphi_{1}SSB_{t-1}+\varphi_{T}T_{t}+\varphi_{T^{2}}T_{t}^{2}}$$ (3)

The dynamics of age classes *s* = 2,…,*n* follow as

$$x_{s+1,t+1}=\alpha_{s}\;\left(1-q_{s}(\sigma_{t})\frac{H_{t}}{B_{t}}\right)x_{st},\;s=1,\ldots ,n-2$$ (4)

$$x_{n,t+1}=\alpha_{n-1}\;\left(1-q_{n-1}(\sigma_{t})\;\frac{H_{t}}{B_{t}}\right)\;x_{n-1,t}+\alpha_{n}\;\left(1-q_{n}(\sigma_{t})\;\frac{H_{t}}{B_{t}}\right)\;x_{n,t}.$$ (5)

In Eqs (4) and (5) $H_{t}=\sum_{s=1}^{n}\;‍w_{s}h_{st}$ denotes total biomass caught (across all age classes), and $B_{t}=\sum_{s=1}^{n}\;‍w_{s}q_{s}(\sigma_{t})x_{st}$ is the efficient stock size ICES [48], that is the total fishable biomass when sorting grid spacing *σ*_*t*_ may limit the age-specific catchabilities *q*_*s*_(*σ*_*t*_) to values below one. The sorting grid became mandatory in the Northeast Arctic in 1997. All fishing trawlers must use a grid with a minimum bar spacing of 55 mm [53]. The equality

$$h_{st}=q_{s}(\sigma_{t})\;\frac{H_{t}}{B_{t}}\;x_{st}$$ (6)

used above follows from the assumption that the share of age class *s* in a catch equals its share in efficient biomass,

$$\frac{w_{s}h_{st}}{H_{t}}=\frac{w_{s}q_{s}(\sigma_{t})x_{st}}{B_{t}}.$$ (7)

The general functional relationship between sorting grid spacing *σ*_*t*_ (in mm) and the age-specific catchability coefficients *q*_*s*_(*θ*_*t*_) are as found in [54]:

$$q_{s}(l_{s})=\frac{e^{a+bl_{s}}}{1+e^{a+bl_{s}}}\;with\;L_{50}=-\frac{a}{b},SR=\frac{\log\left(9\right)}{b}.$$ (8)

For Eq (8) Sistiaga [53] have estimated the following relationships for Northeast Arctic cod, while we use the means of the values reported by ICES [48: tables A5, A7 and A1] for the length-at-age values *l*_*s*_.

$$L_{50}=0.8512\;\sigma_{t}+6.0237$$ (9)

$$SR=4.2079\;e^{0.0125\;\sigma_{t}}$$ (10)

Following the approach of Diekert et al. [55–56] and Diekert [57], this study considers age- and year-specific average landing prices *p*_*s*_, calculated from the landing tickets of Norwegian trawlers. For each weight category and year, the cumulated catch value in NOK was divided by the respective cumulated live weight caught. Our model of fishing costs assumes that fishers choose the different input variables (capital, labor, fuel) such as to maximize profits. Adopting the standard formulation $\Pi =pH-\frac{cH}{X^{\gamma }}$ from biomass models, fishing profits and hence profit margins can be written as

$$\Pi_{t}=\sum_{s=1}^{n}\;‍p_{s}\;w_{s}\;h_{st}-c_{0}\;\frac{H_{t}}{B_{t}^{\gamma }}$$ (11)

$$\pi_{t}=\frac{\sum_{s=1}^{n}‍p_{s}w_{s}q_{s}(\sigma_{t})\frac{H_{t}}{B_{t}}x_{st}-c_{0}\frac{H_{t}}{B_{t}^{\gamma }}}{\sum_{s=1}^{n}‍p_{s}w_{s}q_{s}(\sigma_{t})\frac{H_{t}}{B_{t}}x_{st}},$$ (12)

which we rearrange to yielding the following equation:

$$c_{0}\;B_{t}^{1-\gamma }=(1-\pi_{t})\;\sum_{s=1}^{n}\;p_{s}\;w_{s}\;q_{s}(\sigma_{t})\;x_{st}.$$ (13)

Using time-series data on fish prices, landings, biomass, stock numbers at age, and profit margins, we can use (13) in logarithmic form to estimate the parameters c_0_ and γ of the cost function. For this, annual means of the profit margins of Norwegian trawlers, defined as $\pi_{t}\equiv {\left(\frac{Profit}{Revenue}\right)}_{t}$, were calculated from data of the Norwegian Directorate of Fisheries [58]. The estimates (standard errors in parentheses) of ln(*c*_0_) = 2.8463 (1.1514) and 1−γ = 0.8959 (0.1796) were computed using an Ordinary Least Squares regression with seven data points between 2004 and 2010 (R^2^ = 0.8327). Transforming these estimates yielded *c*_0_ = 17.224 and γ = 0.104.

In our optimization model, we determine catches and grid size of the fishing gear such as to maximize the annual profit (Eq 11) subject to the age-structured population dynamics (Eqs 2–6) and gear selectivity (Eqs 7 and 8). As the population dynamics of cod are on a much faster time scale than ocean warming and acidification, we report all optimized variables for a fish population that is in steady state under the given climate conditions.

### Combined effect of ocean warming and acidification

As the first step of the analysis, we construct a risk of stock collapse indicator by considering the combined effects of fishing, warming and acidification. For this sake, we vary both fishing mortality and January temperature to study the combined effects of fishing and warming on the expected stock size in steady state. We repeat the analysis for the case with and without acidification-induced mortality of early life history stages of cod. Thus, our analysis includes a temporal evolution of January temperature from the Kola section, but can only capture the same level of ocean acidification for every incremental increase in ocean temperature. In all computations, we keep the sorting grid size at 55mm, according to current regulation [53]. Specifically, the risk of stock collapse indicator is constructed by using the age-structured fishery model to compute the resulting steady-state stock size under the combined stressors of ocean warming and acidification and divide it by the unfished steady-state biomass for the optimal temperature. Thereby we obtain an indicator of how the combined stressors of fishing, warming and acidification reduces the equilibrium stock size. The risk of stock collapse is then calculated as one minus this indicator.

As a second step, we used the age-structured ecological-economic optimization model to calculate the economically optimal fishing management in terms of fishing mortality and gear choice for different levels of temperature increase, and with or without acidification effects. We thereby investigate how to best adapt the management of the Northeast Arctic cod fishery to changing environmental conditions. Adaptation to the environmental stressors of ocean warming and acidification could be implemented by reducing fishing pressure on the stock, for example by decreasing total allowable catches, or by adapting the selectivity of fishing gear, to preserve more older spawners which have a higher fertility. We analyze the effect of increasing seawater temperature and fishing pressure on the target stock size. Subsequently, we take the additional mortality due to projected end of century ocean acidification into consideration. Specifically, we calculate the economically optimal fishery management in terms of fishing mortality and gear choice for different levels of temperature increase, and with and without the additional ocean acidification effect on mortality.

## Results

### Risk of stock collapse under fishing, warming and acidification

**Fig 1:**
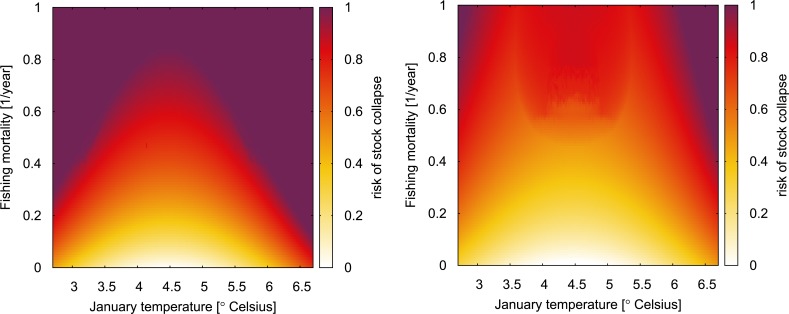
**‘Burning ember’ plots showing the combined effect of fishing and warming on the Northeast Arctic cod stock without taking into account mortality due to acidification (left panel) and the combined effect of fishing and warming plus the extra mortality due to acidification (right panel).** In both plots, the January seawater temperature ranges from 2.7°C (the minimum in the time series) to 6.7°C (beyond the historical maximum) and fishing mortality ranges from zero to one. Color codes show the risk-of-collapse-indicator, with darker shades of red/purple indicating a higher risk of stock collapse.

Fig 1 shows the risk of stock collapse as a function of (i) seawater temperature increase in January, which is the month with the highest explanatory power on recruitment, and (ii) fishing mortality. Darker shades of red indicate a higher risk of collapse. For example, a value of 0.9 translates into a stock decline to 10% of the un-fished biomass at optimal January seawater temperature conditions. While the left panel considers only fishing pressure and ocean warming, the right panel takes the additional effect of larvae mortality due to ocean acidification into consideration, indicating a strongly increased risk of collapse.

**Fig 2:**
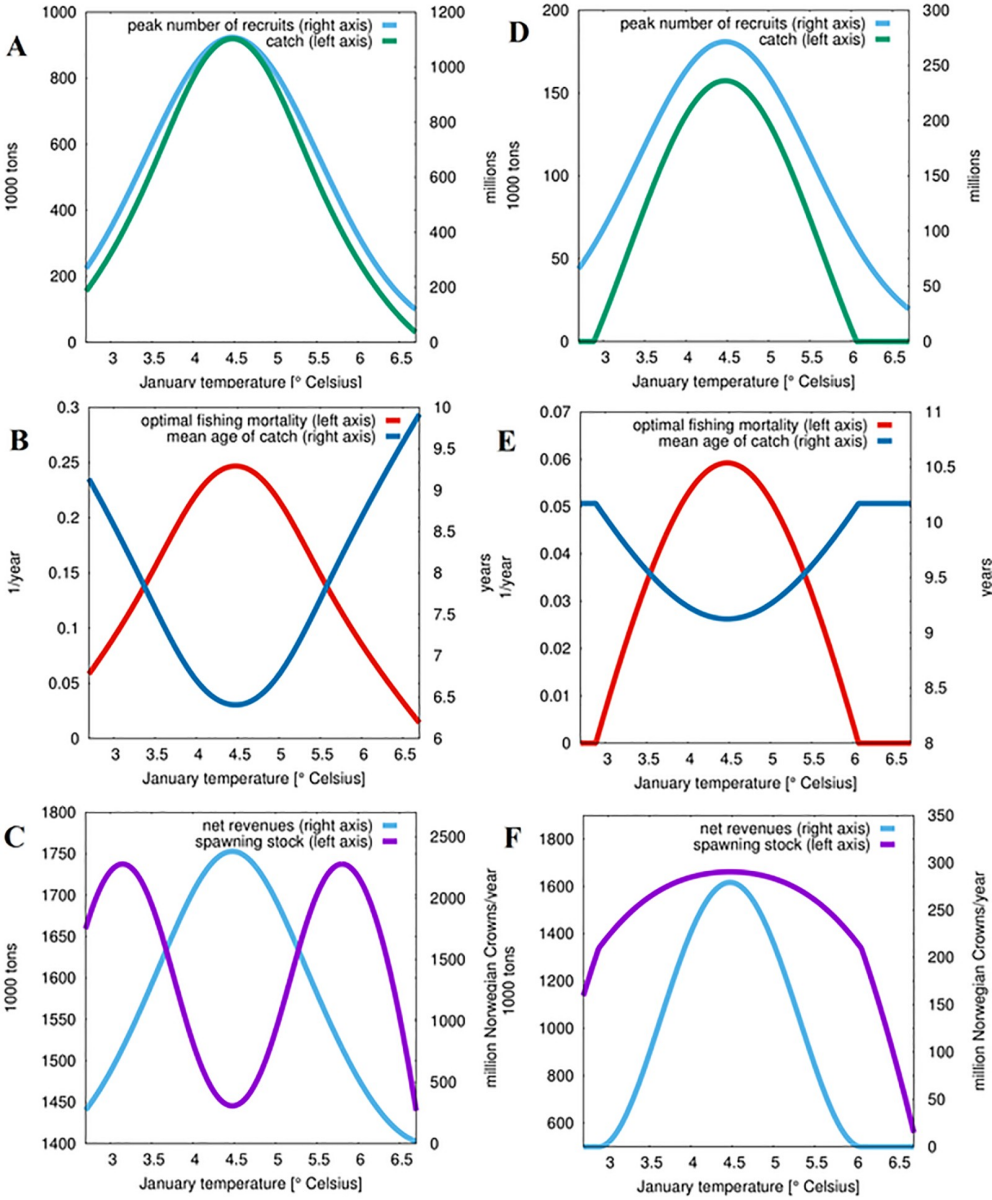
Temperature effects on the optimal management of Northeast Arctic cod (Gadus morhua) with and without the effect of acidification. Depicted are catch and recruits (top panel), fishing mortality and mean age of catch under optimal management (middle panel), and net revenue and spawning stock biomass (lower panel). Plots in the left column show results for warming only, while plots in the right column show results for the combined effect of warming and acidification. It is important to note the marked change in scale for the two sets of vertical axes.

In both panels of Fig 2 an ‘optimal’ temperature of 4.5°C is roughly visible. At this temperature, the stock can support a much higher fishing mortality for the same risk of stock collapse. Without acidification effects (left panel), even at a fishing mortality of *F = 0*.*5* corresponding to a yearly fraction of 39% of the stock fished, the steady-state population size is about half that of the unfished size. Overall, ocean acidification greatly increases the risk of stock collapse. Under the combined effect of warming and acidification, the levels of fishing mortality that the stock can support to minimize the risk of stock collapse are much lower than under warming only. In this case the fishery management would have to adapt by drastically lowering fishing pressure on the stock.

### Socio-ecological effects of ocean warming

The ecological-economic model determines optimized fishing mortality, size of the spawning stock, catch level, and profits for the fishery (Fig 2A–2C). The temperature range considered spans 2.7°C to 6.7°C January temperature. The number of recruits is at a maximum of 1100 million for a temperature optimum around 4.5°C. At about 0.9 million tons, the corresponding catch is close to the levels reached in the early 1970s. This optimal temperature maximizes net revenues at around NOK 2300 (US$ 266) million per year, while the spawning stock biomass is kept at an optimal level of around 1.5 million tons that is only slightly above the current estimated stock level at 1.486 million tons [59]. At the optimal temperature of 4.5°C fishing effort reaches a maximum of 0.25 per year. The sorting grid size is chosen such that the mean age of catch of 6.5 years is close to the current level.

### Socio-ecological effects of ocean warming and acidification

The combined effect of ocean warming and acidification on the stock-recruitment of Northeast Arctic cod results in severe outcomes for the fishery (see Fig 2D–2F). Recruitment and catch are reduced to about a quarter of the numbers reached when acidification is excluded. Under the optimal January seawater temperature for the fishery (4.5°C), the optimal spawning stock biomass is higher and decreases faster under the combined case than under ocean warming only in order to buffer the adverse effects of ocean acidification. Hence, under ocean acidification the fishery is much more vulnerable to temperatures that deviate from the optimum. Net revenues decrease to about 10% of those attained without acidification, while the optimal fishing mortality is reduced by about 80%. In summary all social-ecological indicators point in the same direction: Commercial fishing activities will need to be reduced drastically to sustain the profitability of the Northeast Arctic cod fishery under ocean warming and acidification. As a consequence, it is likely that not only the fishery’s contribution to food security will be at stake, but also less fishermen will be employed in the industry.

## Conclusions and discussion

This paper uses a combination of time-series econometrics, in-situ experiments and an bio-economic optimization model to study the ecological and economic effects of ocean warming and acidification on one of the commercially important fish stocks in the North Atlantic; a natural environment that can be deemed an ‘early warning system’ for climate change in marine environments. The analysis examined how increasing temperature and CO_2_ affect ecological (stock size), economic (profits), consumer-related (harvest) and social (fishing effort) indicators, ranging from present-day conditions to future climate change scenarios. Results show that climate change will benefit the fishery as long as the temperature is still below the optimum for cod reproduction. However, under a combined scenario of ocean warming and acidification that is likely to occur by the end of the century in the Barents Sea, this commercially important fishery may be at risk of collapse, even with the best adaptation efforts in terms of reduced fishing intensity. Our results thus highlight the immediate need for adaptive fisheries management in the light of anticipated ocean warming and acidification in order to sustain the fishery’s profitability, contribution to food security and employment possibilities.

This study faces limitations in current knowledge that can provide new challenges for future research. The first limitation relates to the physiological response of fish populations to ocean acidification, which is highly uncertain. On the one-hand potential negative impacts have been found for the organ structure of larvae fish [15, 17], hatching success [60], sensory abilities and behavior [61–62] or with respect to the survival in very early larval stages [13, 63]. Early life stages prior to gill formation have a limited capacity for pH regulation [64] and are therefore more vulnerable to environmental change. On the other hand, for different fish populations other studies have reported no impact on egg or larval survival under acidification levels addressed in this study [15, 19, 63]. Moreover, research that suggests effects on behavior could not be replicated in recent studies [65]. In addition, due to the high capacity of adult fish to osmoregulate, they have been found to be tolerant to extreme values of ocean acidification [66]. Thus, we neglect the potential for acclimation and adaption to ocean acidification resulting in potentially over-pessimistic scenarios.

The second limitation relates to how ocean warming and acidification are incorporated into the integrated bio-economic modelling framework. With respect to ocean acidification this study builds on the experiments reported by Stiasny et al. [14] that only rely on two CO_2_ scenarios. The elevated CO_2_ treatment corresponds to a business-as-usual emissions scenario consistent with RCP8.5 globally, but is also contained in the confidence intervals for more optimistic scenarios like RCP4.5 and RCP2.6 [4]. Additionally, considering the faster climate change in the Arctic region due to the feedback related to retreating sea ice and the high solubility of CO_2_ in colder waters, ocean acidification in high latitude regions will far exceed the global average making our scenario likely much earlier [67]. Because until now experimental work cannot provide data for a case between the two extreme scenarios, it will be important to explore other ways of quantifying the implications of ocean acidification on recruitment.

Ocean warming, in turn, is a widely accepted driver of vital rates of fish such as growth, or mortality [68–69], while the cod stock’s reaction to temperature increase can be both positive and negative [37]. Thereby, the effect of temperature increase on recruitment has both a direct physiological impact on juveniles and an indirect effect through other climate variables as well as changes in prey fields and food availability [70]. Sundby [70], for example, suggests that the recruitment-temperature relationship for Atlantic cod is a proxy for food availability of the copepod *Calanus finmarchicus* during early life stages. In this paper, we did not describe mechanistic processes how temperature affects recruitment. Rather we have adopted a reduced-form approach and estimated the compound effect of temperature changes on cod recruitment by means of a statistical analysis of time series data. Accordingly, projections have to be considered with caution, especially if they go beyond the past range of variation in climate variables and recruitment.

Moreover, this study relies on the monthly-integrated mean temperature for 0–200 m depth along the Kola Section, as well as general scenarios for atmospheric CO_2_ concentrations and hence lacks the spatial resolution relevant for cod spawning areas. The Kola section temperature in the eastern Barents Sea does not fully reflect the ambient temperature that the recruiting larvae and pelagic juveniles experience along the drift pattern during the first half year of life. Moreover, this study abstracts from spatial redistribution of the cod stock due to changing environmental conditions [37]. One promising research approach that overcomes the limitation on how ocean warming and acidification are incorporated into the modeling framework could be to use an ecosystem model that explicitly captures both *in situ* warming and acidification for different depth layers in the Barents Sea. Such a model could also take into account the availability of food as an important driver of recruitment and hence cover direct, indirect and combined effects.

Finally, our study has focused on the long-term average effects of ocean warming and acidification. In reality, fish populations commonly exhibit substantial variability in recruitment, even under present-day climate conditions [e.g. 71–72]. Whereas previous studies suggest that the optimal management strategy is not sensitive to the stochastic nature of recruitment [52], a stochastic model that fully takes into account the variability in recruitment by considering a stochastic optimization would be needed to more precisely assess the effects of ocean warming and acidification on the likelihood of stock collapse, especially on shorter time scales.

In summary, the modelling approach described here cannot capture all real world complexities associated with the socio-ecological effects of ocean warming and acidification on an important commercial fishery, such as that for Northeast Arctic cod. On this basis, rather than considering the results obtained here as firm predictions, they should be seen as an illustration of what could happen if unregulated ocean acidification was added to the well-known stressors of warming and fishing.

## Code availability

The ecological-economic optimization model is implemented in the AMPL programming language [73] and solved using Knitro 10.3. Programming codes that list all remaining age-specific parameter values are available on request by the authors of this study.

## Supporting information

S1 Table Parameter values used in the age-structured fishery model. (DOCX)
