# Supplementary material for: Ocean warming and acidification may drag down the commercial Arctic cod fishery by 2100
Source: PLoS One. 2020 Apr 22;15(4):e0231589. doi: 10.1371/journal.pone.0231589 (PMC7176117; doi:10.1371/journal.pone.0231589)
Supplement: S1 Table — (DOCX) [file pone.0231589.s001.docx]

**S1 Table. Parameter values used in the age-structured fishery model.**

| Age | Weight (kg) | Price (NOK/kg) | Survival | Maturity | Catchability (mesh size 110mm) |
| --- | --- | --- | --- | --- | --- |
| 1 | 0 | 0 | 1 | 0 | 0 |
| 2 | 0 | 0 | 1 | 0 | 0 |
| 3 | 0.75 | 11.48 | 0.82 | 0 | 0 |
| 4 | 1.18 | 11.48 | 0.82 | 0 | 0 |
| 5 | 1.68 | 12.22 | 0.82 | 0.06 | 0 |
| 6 | 2.32 | 12.22 | 0.82 | 0.32 | 0.01 |
| 7 | 3.19 | 12.22 | 0.82 | 0.64 | 0.02 |
| 8 | 4.38 | 14.12 | 0.82 | 0.85 | 0.06 |
| 9 | 5.83 | 14.12 | 0.82 | 0.95 | 0.18 |
| 10 | 7.32 | 16.56 | 0.82 | 0.98 | 0.39 |
| 11 | 8.8 | 16.56 | 0.82 | 0.99 | 0.52 |
| 12 | 10.48 | 16.81 | 0.82 | 1 | 0.68 |
| 13 | 13.11 | 16.8 | 0.82 | 1 | 0.68 |
